# DMP-1 attenuates oxidative stress and inhibits TGF-**β** activation in rats with diabetic kidney disease

**DOI:** 10.1080/0886022X.2016.1256319

**Published:** 2016-11-23

**Authors:** Na Du, Shunan Liu, Chongshuang Cui, Mo Zhang, Jibin Jia, Xia Cao

**Affiliations:** a Department of Pharmacology, Jilin University, Changchun, Jilin, China;; b Department of Burn Surgery, The First Hospital of Jilin University, Changchun, Jilin, China

**Keywords:** DMP-1, oxidative stress, TGF-β, diabetic kidney disease, diabetes mellitus

## Abstract

**Introduction:** DMP-1 supplement has a satisfactory effect on diabetic kidney disease in patients with whether T1DM or T2DM. Oxidative stress and TGF-β signal pathway activation are essential in the pathogenesis of DKD. We aim to investigate the effect of DMP-1 on oxidative stress and TGF-β activation in rats with DKD.

**Materials and methods:** Male Wistar rats were enrolled and randomly allocated into five groups: Control group, STZ group (60 mg/kg, ip), DMP-1 low dose group (0.5 g/kg/day, ig), DMP-1 medium dose group (1.0 g/kg/day, ig) and DMP-1 high dose group (2.0 g/kg/day, ig). The levels of UREA, BUN, UCr, β_2_-MG, mALB, NOS, CAT, MDA and T-AOC were measured after 8 weeks treatment. And rats’ left kidneys were harvested to detect the expression of TGF-β, Smad2/3 and Smad7 by immunohistochemical analysis.

**Results:** DMP-1 treatment has protective effects on kidney injury induced by STZ, which is demonstrated as following criteria: (1) a significant reduction in levels of UREA (*p* < 0.05), BUN (*p* < 0.05), UCr (*p* < 0.05), β2-MG (*p* < 0.05) and mALB (*p* < 0.05) in rats treated by DMP-1 compared with the ones injected with STZ only; (2) an apparent increment levels of NOS (*p* < 0.05), CAT (*p* < 0.05) and T-AOC (*p* < 0.05), while reduction in level of MDA (*p* < 0.05) in DMP-1 groups compared with STZ group; (3) a significant inhibition of TGF-β and Smad2/3 overexpression induced by STZ in kidney tissue. What’s more, DMP-1 can increase Smad7 expression.

**Conclusion:** DMP-1 could slow pathological process and protect kidney from DKD injury by decreasing oxidative stress and inhibiting TGF-β signal pathway activation in rats.

## Introduction

Diabetic kidney disease (DKD) as one of the severe complications of diabetes mellitus (DM) has always been the most common cause of end-stage renal failure (ESRF) nowadays.^1^ Although the plasma glucose levels can be strictly controlled by insulin or other hypoglycemic medicine, there are still 30% to 40% DM patients suffering from DKD at last.^2^^,^^3^ The therapeutic regimen that managing the hyperglycemia and hypertension is employed in clinic can reduce the proportion of DM patients reaching ESRF.^4^ But there is still lack of additional and effective therapies and medicines to the ones with progressive DKD.^5–8^ The clinical data show that there is a rapidly rising of DM in China and then DKD incidence will reach a peak which is enough to be regarded as a public health concern for decades to come.^9^^,^^10^ It is vital to find out novel therapies or medicines to improve and treat DKD.

Characterized by glomerular hypertrophy, infiltration of inflammatory factors, accumulation of extracellular matrix and renal fibrosis, DKD which is far from complete of our understanding, involves in many complex disease processes.^11–13^ Such as lipoprotein metabolic abnormalities, oxidative stress and transforming growth factor-β (TGF-β)/Smad signal pathway activation are contributed to DKD’s development and progression.

Many experiments results have showed that oxidative stress is important to develop and persistently progress DKD. It has been certified that metabolic abnormalities induced by DM can produce a large amount of redundant reactive oxygen species (ROS) and advanced glycation end products (AGEs).^14^^,^^15^ ROS and AGEs as the central and major mediators of microvascular injury will trigger many cell signal pathways activation in charge of DKD such as TGF-β/Smad pathway which speeds up the process of renal fibrosis. Herein, decrease of ROS and inhibition of TGF-β/Smad signal pathway activation in kidney may be the target for new drugs.

DMP-1 supplement for DM patients has been used in Chinese traditional medicine clinic to help stabilize their blood glucose homeostasis and improve their complication. Some previous experimental findings and data that come from clinical statistical analysis show that DMP-1 has an effective protection from DKD or delay its progression. Although the precise mechanisms of DMP-1 are unclear, DMP-1 can decrease ROS such as H_2_O_2_ and increase parameters of renal function (the work have been finished in our group).

Here, we explored the effect of DMP-1 on oxidative stress and TGF-β activation in DKD rats to seek its potential mechanism.

## Materials and methods

### Chemicals

DMP-1 (named Yishenhuoxue granule) was a traditional Chinese herbal compound as clinical prescription. And it was composed of ginsengradixetrhizoma, radix astragali, fructus schisandrae chinensis and other three medicinal materials. Rat UREA, blood urea nitrogen (BUN), urinary creatinine (UCr), β_2_-microglobulin (β_2_-MG) and microalbuminuria (mALB) ELISA kits were purchased from Jiancheng Bioengineering Institute, Nanjing, China. The NOS, catalase (CAT), methane dicarboxylic aldehyde (MDA) and total antioxidative capacity (T-AOC) assay kits were bought from Jiancheng Bioengineering Institute as well. STZ was purchased from Sigma, while polyclonal antibody of TGF-β, Smad2/3 and Smad7 were purchased from Santa Cruz Biotechnology. Rat SP immunohistochemistry kits were purchased from Invitrogen. The other medicine reagents were of analytical grade.

### Animals

60 male Wistar rats (180–220 g) were purchased from Laboratory Animal Center of Jilin University. All animal experiments were permitted by the Animal Care Committee of Jilin University Pharmaceutical College. The rats were kept under standard surroundings with room temperature of 22 ± 2 °C, 12 h/12 h light/dark cycle and free to food and water.

### *In vivo* experiments

After acclimating to the facilities for one week, the rats were randomly divided into five groups (*n* = 10) as follow: (1) Control group (vehicle injection), (2) STZ group (rats were given 60 mg/kg of STZ within 0.1 mol/L sodium citrate solution for pH 4.50, ip), (3) DMP-1 low dose group (STZ-injection rats were given 0.5 g/kg/day of DMP-1, ig), (4) DMP-1 medium dose group (STZ-injection rats were given 1.0 g/kg/day of DMP-1, ig, equivalent to the clinical dose) and (5) DMP-1 high dose group (STZ-injection rats were given 2.0 g/kg/day of DMP-1, ig). The blood glucose levels of the rats except ones in control group were measured at 72 h after STZ-injection. Only the ones with blood glucose concentration higher than 13.8 mmol/L had been employed in this study.

At the end of 8 weeks, the blood and 24 h urine samples were collected to detect the levels of UREA, BUN, UCr, β_2_-MG, mALB, NOS, CAT, MDA and T-AOC by assay kits according to the manufacturer's instructions. After euthanized, kidneys (left) were harvested and rinsed free from blood with PBS. Fixed in 10% neutral formalin and embedded in paraffin, the antigen of kidney tissue slides was exposed by treatment with boiling citrate buffer (0.01 mol/L, pH 6.0). Then the slides were respectively incubated with TGF-β, Smad2/3 and Smad7 antibody for immunohistochemical analysis according to the manufacturer's instructions and examined by a light microscope (Nikon Ti). The analyzes were performed to test for the gray value of the immunohistochemical staining using Motic Images Advanced 3.2.

### Statistical analysis

Statistical analyzes were performed utilizing SPSS 20.0 programs. Data were presented as means ± standard deviations (SDs) and analyzed using One-way ANOVA and LSD, *post hoc*. And overall statistical significance was determined at *p* < 0.05.

## Results

### DMP-1 attenuates kidney injury induced by DKD

**Figure 1.**
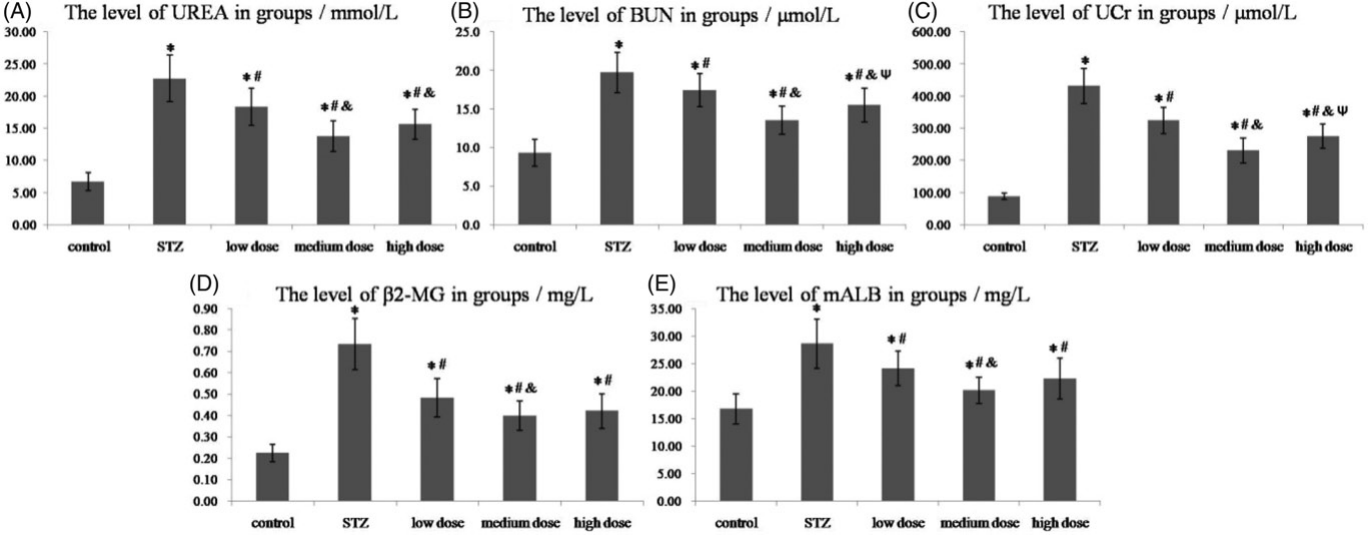
The levels of UREA, BUN, UCr, β_2_-MG and mALB in groups. (A) The plasma level of UREA; (B) The plasma level of BUN; (C) The urine level of UCr; (D) The urine level of β_2_-MG; (E) The urine level of mALB. *p < 0.05 vs. control group; #*p* < 0.05 vs. STZ group; &*p* < 0.05 vs. DMP-1 low dose group; Ψ*p* < 0.05 vs. DMP-1 medium dose group.

Many experiments have demonstrated that the factor levels of UREA, BUN, UCr, β_2_-MG and mALB are increased in STZ-induced DKD.^16–18^ And our results also showed that STZ could induce a significant increase of these factor levels compared with control group (shown in Figure 1 and Table 1, *p* < 0.05). From Figure 1(A), we knew that DMP-1 could significantly decrease the level of UREA after 8 weeks treatment in DMP-1 low dose group, DMP-1 medium dose group and DMP-1 high dose group (*p* < 0.05 vs. STZ). However, rats in DMP-1 medium dose group had the lowest level of UREA within the three groups (*p* < 0.05 vs. DMP-1 low dose group; *p* > 0.05 vs. DMP-1 high dose group).

**Table 1.** The levels of urea, BUN, UCr, β_2_-MG and mALB in groups (*n* = 10).

Factors	Control	STZ	DMP-1 low dose	DMP-1 medium dose	DMP-1 high dose
UREA/mmol/L	6.71 ± 1.36	22.76 ± 3.66	18.35 ± 2.91	13.75 ± 2.36	15.60 ± 2.37
BUN/μmol/L	9.3 ± 1.7	19.7 ± 2.6	17.5 ± 2.1	13.6 ± 1.8	15.5 ± 2.2
UCr/μmol/L	88.41 ± 10.02	432.43 ± 54.60	324.29 ± 41.25	230.38 ± 38.54	275.56 ± 37.66
β_2_-MG/mg/L	0.22 ± 0.04	0.73 ± 0.12	0.48 ± 0.09	0.40 ± 0.07	0.42 ± 0.08
mALB/mg/L	16.72 ± 2.74	28.56 ± 4.48	24.09 ± 3.16	20.07 ± 2.39	22.25 ± 3.70

At the end of 8 weeks, DMP-1 treatment could decrease the level of BUN (shown in Figure 1(B)), *p* < 0.05 vs. STZ). Compared with DMP-1 low dose group, DMP-1 medium dose group and DMP-1 high dose group had lower BUN levels (*p* < 0.05). And DMP-1 medium dose group had lower level of BUN than DMP-1 high dose group (*p* < 0.05).

Simliar to BUN, rats in DMP-1 groups had lower levels of UCr compared with the ones in STZ group (*p* < 0.05). DMP-1 medium dose group and DMP-1 high dose group had lower UCr levels (*p* < 0.05 vs. DMP-1 low dose group). And DMP-1 medium dose group had significantly decreased the UCr level than DMP-1 high dose group (*p* < 0.05) (Figure 1(C)).

As shown in Figure 1(D), DMP-1 could significantly decrease the rise of β_2_-MG levels induced by STZ-injection. DMP-1 medium dose group had the lowest level of β_2_-MG in the DMP-1 groups (*p* < 0.05 vs. DMP-1 low dose group or DMP-1 high dose group). But there was no significant difference between DMP-1 low dose group and DMP-1 high dose group.

The Figure 1(E) indicated that, the three DMP-1 groups significantly attenuated mALB levels compared with STZ rats (*p* < 0.05). And DMP-1 medium dose group had the lowest level of mALB in the DMP-1 groups (*p* < 0.05 vs. DMP-1 low dose group or DMP-1 high dose group).

### DMP-1 decreases oxidative stress induced by DKD

**Figure 2.**
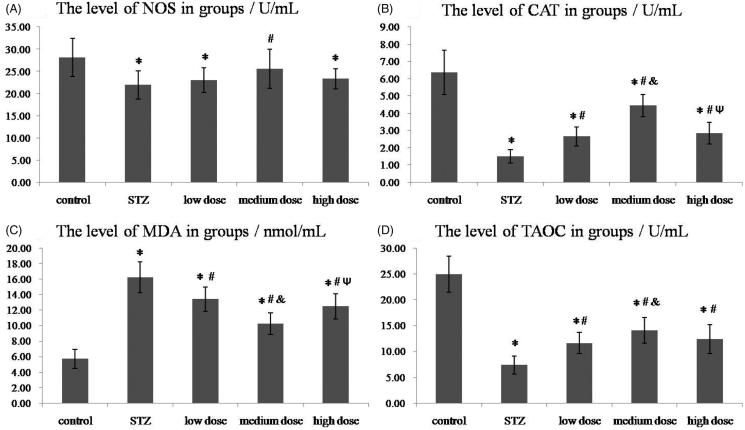
The levels of NOS, CAT, MDA and T-AOC in groups. (A) The plasma level of NOS; (B) The plasma level of CAT; (C) The plasma level of MDA; (D) The plasma level of T-AOC. *p < 0.05 vs. control group; #*p* < 0.05 vs. STZ group; &*p* < 0.05 vs. DMP-1 low dose group; Ψ*p* < 0.05 vs. DMP-1 medium dose group.

Shown in Figure 2 and Table 2, STZ induced an obvious decrease of anti-oxidative stress factors (NOS, CAT and T-AOC) compared with control group and induced a significantly increase of oxidative stress factor such as MDA. As Figure 2(A) showed, DMP-1 medium dose treatment could significantly raise NOS level (*p* < 0.05 vs. STZ). Although the DMP-1 low dose group and DMP-1 high dose group had higher levels of NOS, there was no statistical difference with STZ group (*p* > 0.05).

**Table 2.** The levels of NOS, CAT, MDA and T-AOC in groups (*n* = 10).

Factors	Control	STZ	DMP-1low dose	DMP-1medium dose	DMP-1high dose
NOS/U/mL	28.15 ± 4.33	21.94 ± 3.24	23.07 ± 2.80	25.57 ± 4.42	23.32 ± 2.31
CAT/U/mL	6.37 ± 1.29	1.50 ± 0.38	2.66 ± 0.56	4.44 ± 0.65	2.85 ± 0.64
MDA/nmol/mL	5.71 ± 1.21	16.19 ± 1.99	13.39 ± 1.56	10.25 ± 1.39	12.46 ± 1.61
T-AOC/U/mL	24.92 ± 3.47	7.35 ± 1.72	11.60 ± 2.06	14.09 ± 2.47	12.37 ± 2.80

DMP-1 treatment could increase the level of CAT (shown in Figure 2(B), *p* < 0.05 vs. STZ). Compared with DMP-1 low dose group, DMP-1 medium dose group had higher CAT level (*p* < 0.05). And DMP-1 medium dose group also had higher CAT level than DMP-1 high dose group (*p* < 0.05).

For MDA factor, DMP-1 treatment could inhibit the level raise in plasma (shown in Figure 2(C), *p* < 0.05 vs. STZ). Compared within the three DMP-1 groups, DMP-1 medium dose group had the best depressor effect (*p* < 0.05 vs. DMP-1 low dose group or DMP-1 high dose group). However, there was no statistical difference between DMP-1 low dose group and DMP-1 high dose group as *p* > 0.05.

**Figure 3.**
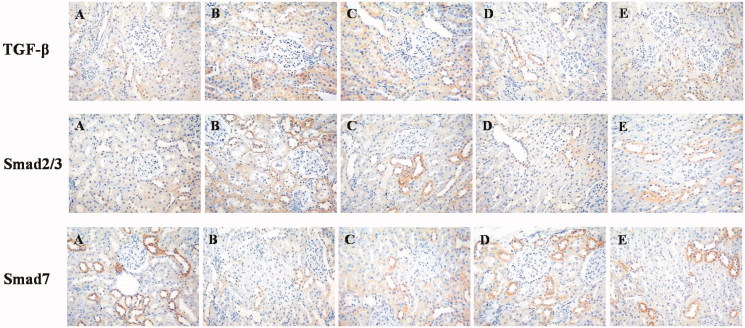
Photomicrographs of representative immunohistochemical staining sections of kidney (200× magnification). (A) Control group; (B) STZ group. (C) DMP-1 low dose group; (D) DMP-1 medium dose group; (E) DMP-1 high dose group.

In Figure 3(D), we could conclude that DMP-1 treatment had improved the total antioxidant capacity in rats after STZ-injection apparently (*p* < 0.05 vs. STZ). And rats in DMP-1 medium dose group had better improvement of T-AOC than the other two DMP-1 groups.

### Immunohistochemical staining of TGF-β, Smad2/3 and Smad7 in groups

**Figure 4.**
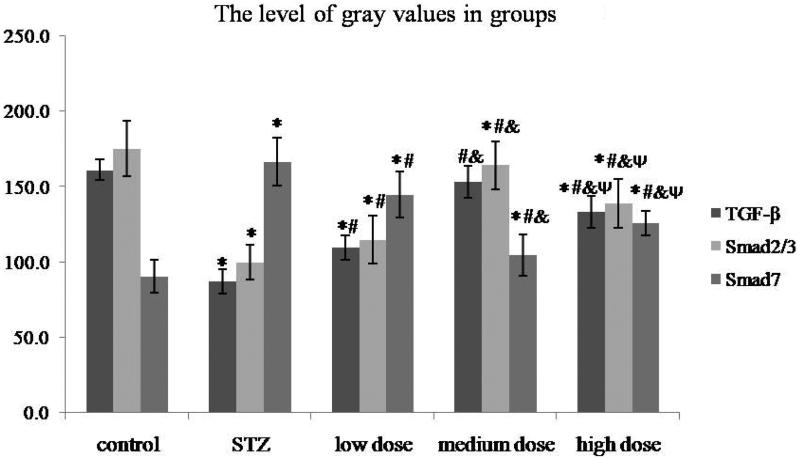
The gray values of TGF-β, Smad2/3 and Smad7 in groups. *p < 0.05 vs. control group; #*p* < 0.05 vs. STZ group; &*p* < .05 vs. DMP-1 low dose group; Ψ*p* < 0.05 vs. DMP-1 medium dose group.

In order to assess the effect of DMP-1 on TGF-β activation in rat’s kidney, the immunohistochemical staining of TGF-β, Smad2/3 and Smad7 were performed. Compared with control group, rats in STZ group showed sever immunopositivity for TGF-β and Smad2/3, while no immunopositivity for Smad7 (Figure 3(B)). However, the DMP-1 treatment could decrease immunopositivity for TGF-β, Smad2/3 and increase immunopositivity for Smad7 (Figure 4). The more vital was that DMP-1 medium dose group had the lowest levels of TGF-β, Smad2/3 and the highest level of Smad7 within three DMP-1 groups by gray value test of immunohistochemical staining using Motic Images Advanced 3.2 (shown in Figure 4; the severer immunopositivity was, the lower gray value tested).

## Discussion

As the most common and high incidence comorbidity of DM, DKD is a clinical syndrome with early glomerular hyperfiltration and albuminuria, developing into increasing proteinuria, a decline in glomerular filtration rate and renal fibrosis.^19^ It is alarming that DKD is prevalent and becoming to be the leading cause of ESRF followed by DM incidence increasing.^20^^,^^21^ Indeed, diabetes mellitus induces all structural compartments’ functional impairments in patient’s kidney.^12^ The understanding of pathogenesis, correlation between histological lesions and progression of clinical DKD has been shaped owed to continuous basic science and clinical research. Proposed by Renal Pathology Society,^22^ the pathology classifies of DKD as: Grade 1- is the GBM injury with two standard deviations thickening than normal, Grade 2- is mesangial expansion, Grade 3- is with Kimmelstiel Wilson lesion, Grade 4- is more than 50% glomeruli involved in advanced diabetic glomerulosclerosis in addition to the above changes.

With 60 mg/kg dose intraperitoneal injection of STZ, we can get the DKD animal models whose renal pathophysiological changes and deteriorated functions are similar to those in human beings.^23^ In our study, a single injection of STZ has a success rate over 95% in rats which induces blood glucose concentration higher than 13.8 mmol/L at 72 h after STZ-injection. The levels of urea, BUN, UCr, β_2_-MG and mALB are all significantly increased in STZ group compared with control group (*p* < 0.05) at the end of experiment. We conclude that hyperglycemia induced by STZ can cause DKD injury. The results also show that the plasma levels of NOS, CAT and T-AOC are apparently decreased while MDA level is significantly increased in STZ group (vs. control, *p* < 0.05). Demonstrated by immunohistochemical staining, the TGF-β and Smad2/3 in STZ group rats’ kidneys are overexpressed while Smad7 is low expressed (vs. control, *p* < 0.05).

The oxidative stress and TGF-β/Smad activation are two pivotal pathogenesises in the development of DKD in DM patient cohort.^24–26^ What’ more, they together with other disease processes interactively and rapidly promote DKD. ROS which are the glucose metabolic compounds can be cleaned by antioxidant defense systems and do no harm to organisms under normal physiological conditions. However hyperglycemia producing more ROS beyond clear ability will injure cellular components with oxidative stress that can also induce inflammatory response in kidney tissue. NOS and CAT are the antioxidant enzymes cleaned NO and H_2_O_2_. The increment of MDA reflects the degree of lipid peroxides. And T-AOC can be a significant indicator of compounds potential antioxygenation property.

TGF-β is a major pro-fibrogenic cytokine whose expression is up-regulated in both animal renal fibrosis models and human counterparts.^27^ Experiments have indicated that the transition of renal tubular epithelial cells to myofibroblasts which synthesize excessive amounts of extracellular matrix thus leading to renal fibrosis is modulated by TGF-β through TGF-β/Smad cell signal pathway.^28^ Smad2/3 is phosphorylated stimulated by activated TGF-β receptors. And then turns complexes with Smad 4 which translocate from the cytoplasm into the nucleus regulating the target genes expression.^27^ Smad7 as the opposed manner to Smad2/3 can down-regulate TGF-β signaling.

Many clinical experiences have showed that Chinese herbal compound prescription can protect patients’ kidneys from DKD injury with various and complex mechanisms.^29–31^ In our study, we also found that DMP-1 had protective effects on DKD in rats induced by STZ-injection, which was demonstrated as following criteria: 1) a significant reduction in levels of urea (*p* < 0.05), BUN (*p* < 0.05), UCr (*p* < 0.05), β2-MG (*p* < 0.05) and mALB (*p* < 0.05) in rats treated by DMP-1 compared with STZ group; 2) an apparent increment levels of NOS (*p* < 0.05), CAT (*p* < 0.05) and T-AOC (*p* < 0.05), while reduction in level of MDA (*p* < 0.05) in DMP-1 groups compared with STZ group; 3) a significant inhibition of TGF-β and Smad2/3 overexpression in kidney tissue. And DMP-1 could increase Smad7 expression as well; 4) rats in DMP-1 medium dose group whose dosage were equal to the clinical dose had the best kidney protected effect among the three DMP-1 groups.

DMP-1 composed of six natural Chinese herbals has many advantages of various biological activities and low toxicity. Many DM patients can get some benefits by using it such as a more stabilized blood glucose homeostasis and lower complication incidence. However its potential kidney protection from DKD injury has not taken into account which will limit its clinical usage for shortage of studies and experiment data. The specific mechanisms of DMP-1 on anti-DKD may expand its application. The other therapeutic mechanisms, long-term effects of DMP-1 on DKD and determination of active ingredients will inspire us to do further research.

In conclusion, our findings provide direct evidence for the positive protection of DMP-1 on DKD induced by STZ in rats. We conclude that the DMP-1 can attenuate oxidative stress and inhibit TGF-β activation in DKD. The positive protection offered by DMP-1 medium dose (1.0 g/kg/day) is more effective than DMP-1 low dose or high dose. Therefore, our studies provide both a theoretical basis and laboratory evidence for the DMP-1 application as supplement treatment of patients with DKD. However, more investigations are required to study its mechanism in depth and justify the active constituents which worked on decrease oxidative stress and inhibition TGF-β/Smad cell signal pathway.
